# Sympathetic reactivity to physiological stress is associated with expanded cardiac extracellular volume in humans

**DOI:** 10.1186/s12916-025-04197-6

**Published:** 2025-07-01

**Authors:** Hazel C. Blythe, Zoe H. Adams, Katrina A. Hope, Richard P. Baker, Melanie J. Hezzell, M. Saadeh Suleiman, Ana Paula Abdala Sheikh, Nathan Manghat, Konstantina Mitrousi, Angus K. Nightingale, Emma C. Hart

**Affiliations:** 1 School of Physiology, Pharmacology and Neuroscience, University of Bristol, Biomedical Sciences Building, Bristol, BS8 1TD UK; 2 Department of Cardiology, Musgrove Park Hospital, Somerset NHS Foundation Trust, Taunton, UK; 3 Bristol Veterinary School, University of Bristol, Bristol, UK; 4 Bristol Medical School, University of Bristol, Bristol, UK; 5 Bristol Heart Institute, University Hospitals Bristol and Weston NHS Foundation Trust, Bristol, UK; 6 Cardiff School of Sport and Health Sciences, Cardiff Metropolitan University, Cardiff, UK

**Keywords:** Extracellular volume, Cardiac fibrosis, Sympathetic nerve activity, Stressor

## Abstract

**Background:**

Expanded extracellular volume (ECV) is an early marker of myocardial interstitial fibrosis in patients with hypertension. Animal studies suggest that surges in sympathetic nerve activity (SNA) might contribute more to the development of interstitial fibrosis than the resting level of SNA. The aim of this study was to investigate whether resting SNA or greater SNA reactivity to a stressor may be associated with expanded ECV in humans across a range of blood pressures.

**Methods:**

This was a cross-sectional study in 19 individuals with varying levels of ambulatory systolic blood pressure (111–153 mmHg, 48 ± 13 years, 26.5 ± 2.6 kg/m^2^, *n* = 10 diagnosed with hypertension and *n* = 9 normotensive controls). Beat-to-beat non-invasive blood pressure (Finometer), heart rate (3-lead ECG) and muscle SNA (MSNA; peroneal microneurography) were recorded simultaneously during baseline, and throughout a cold pressor test (physiological stress), with hand immersion in 3–4 °C water. LV chamber size, wall thickness and ECV were assessed using cardiac magnetic resonance imaging.

**Results:**

Resting MSNA was not associated with cardiac ECV (*B* coefficient = − 0.07, 95% CI (− 0.24–0.10), *P* = 0.549), but SNA reactivity to the cold pressor test was a predictor of ECV independent of daytime systolic blood pressure (*B* coefficient = 0.12, 95% CI (0.05–0.20), *P* = 0.007). We determined associations between ECV and MSNA variables using liner regressions, with ECV as the dependent variable.

**Conclusions:**

Our findings show that SNA responses to physiological stress were predictive of ECV, whereas resting SNA was not, independent of the level of blood pressure. Thus, surges in SNA during stress might be more important in cardiac remodelling than overall resting levels of SNA. Further studies should test this hypothesis in larger cohorts.

**Supplementary Information:**

The online version contains supplementary material available at 10.1186/s12916-025-04197-6.

## Background

It is established that in patients with hypertension, presence of left ventricular hypertrophy (LVH) increases cardiovascular morbidity and mortality [1]. However, LVH is also an independent predictor of cardiovascular events in the general population, regardless of resting blood pressure [2]. In addition to myocyte hypertrophy, the myocardium undergoes changes in the extracellular matrix including myocardial interstitial fibrosis, which also contributes to cardiac dysfunction, impairs tissue compliance and reduces the filling capacity of the heart [3].

Myocardial fibrosis is caused by proliferation of fibroblasts, which produce and maintain collagen, forming the extracellular matrix. Collagen provides the structural framework in the healthy myocardium and makes up the extracellular volume (ECV). When an imbalance between collagen fibre formation and degradation occurs, interstitial collagen increases resulting in the expansion of the extracellular matrix. LV ECV, measured using cardiac magnetic resonance imaging in humans, serves as a continuous measurement of interstitial collagen volume and is an early marker of myocardial interstitial fibrosis development [4]. Elevated sympathetic nerve activity (SNA) directed to the myocardium directly contributes to LVH in hypertension, independent of the resting level of blood pressure [5–7]. Numerous studies have demonstrated that the sympathetic nervous system (SNS) plays a significant role in the development of myocardial interstitial fibrosis in animals, which is attenuated with β-adrenergic receptor antagonism [8–10]. Additionally, surgical removal of the superior cervical ganglion in spontaneously hypertensive rats (SHRs) reduced LVH and levels of myocardial fibrosis compared to untreated rats [11].

In humans, resting muscle SNA (MSNA) is higher in individuals with LVH compared to those without LVH, despite similar blood pressure levels [5–7]; however, it is not known whether resting SNA is associated with myocardial interstitial fibrosis. Furthermore, in humans, only resting SNA is considered when examining how the SNS may contribute to cardiac remodelling (LVH). Importantly, humans are not in a constant rested state and are frequently exposed to different physiological stressors throughout the day, resulting in surges in SNA [12]. Intermittent β-adrenergic receptor stimulation with isoprenaline in normotensive mice was found to induce more severe cardiac fibrosis compared to an identical dose of isoprenaline administered continuously [13]. Thus, exaggerated SNA reactivity to stressors may contribute to cardiac remodelling and fibrosis more than the overall level of resting SNA, independent of blood pressure levels.

Therefore, we aimed to determine whether resting SNA and/or SNA reactivity to a physiological stressor (cold pressor test) were associated with ECV in humans with and without hypertension. We hypothesised that resting SNA and the reactivity of SNA to stress would be associated with elevated ECV, irrespective of ambulatory blood pressure levels. If surges in SNA are linked to greater extracellular volumes, regardless of blood pressure levels, this may offer a new therapeutic target for preventing or reversing myocardial interstitial fibrosis in humans, which goes beyond conventional β-blocker therapies.

## Methods

This was a prospective, cross-sectional study.

### Participants

Ethical approval for this study was granted by South-West Bristol NHS Research Ethics Committee (20/SW/0006) and the Health Research Authority, and the study was conducted in accordance with the Declaration of Helsinki (2017). Participants provided written, informed consent to participate in this study. Hypertension was defined as a clinic blood pressure of ≥140/90 mmHg and daytime ambulatory blood pressure of ≥135/85 mmHg, or the use of antihypertensive medication [14]. Participants with hypertension were recruited via (1) a tertiary care Hypertension Clinic (based in University Hospitals Bristol and Weston NHS Trust) and (2) the general public. Normotensive participants were recruited via posters and leaflets around the local area, university newsletters and information on the research group website. All participants were aged 18–65 years old. Inclusion and exclusion criteria are listed in Additional file 1 methods.

### Study visits

Participants were asked to avoid strenuous exercise and refrain from alcohol and caffeine for 12 h prior to each study visit. Autonomic and stress testing was conducted in study visit one at the Clinical Research Facility, Bristol. Cardiac magnetic resonance imaging (CMR) was completed in study visit two at the Bristol Heart Institute.

In visit one, a medical history screening questionnaire was completed, and participants’ height, weight and clinic blood pressures were taken (Omron, 705IT, Omron Healthcare, Kyoto, Japan) following 10 min of seated rest as per European Society of Cardiology/European Society of Hypertension guidelines [15]. Participants were then positioned semi-supine on the bed and fitted with a 3-lead ECG (BioAmp, AD Instruments) to measure heart rate, and Finapres (Finometer Pro, FMS, Netherlands) for beat-to-beat blood pressure. Multi-unit postganglionic sympathetic nerve activity was then recorded using microneurography from the peroneal nerve as previously described [16]. Briefly, following cutaneous electrical stimulation to map out the path of the peroneal nerve, a tungsten microelectrode was positioned to target muscle sympathetic fascicles, and a reference electrode placed 2–3 cm from the recording electrode. A satisfactory signal was confirmed by a pulse-synchronous bursting pattern at a 3:1 signal to noise ratio, and increased MSNA burst frequency with an end-expiratory breath hold [16–18], and no response to startle stimuli. The nerve signal was amplified (total gain 80,000), band-pass filtered (700–2000 Hz) full-wave rectified and integrated at a 0.1 s time constant (Nerve Traffic Analyser, University of Iowa, USA).

MSNA and beat-to-beat heart rate and blood pressure were continuously recorded in LabChart acquisition software (LabChart Pro V7, AD Instruments) throughout a 5–10-min baseline and the cold pressor test (CPT) protocol involving a 2-min rest period followed by 3 min of hand immersion to the wrist in 3–4 °C water. Next, cardiopulmonary exercise testing (CPET) was performed to determine whether the participants’ cardiorespiratory fitness was matched between the normotensive and hypertensive groups (see Additional file 1 for methods). Finally, participants were then fitted with an ambulatory blood pressure monitor (ABPM; Mobil-o-graph, IEM, GmbH, Stolberg, Germany) for 24 h to confirm normotensive or hypertensive status.

A venous blood sample was collected from participants at the time of CMR to measure haematocrit. CMR (1.5 Tesla MR system, Avanto, Siemens, Erlangen, Germany) was performed as previously described [19, 20] to assess cardiac remodelling. Late gadolinium enhancement and native T1 mapping were used to assess levels of replacement and interstitial myocardial fibrosis respectively. Gadobutrol (0.1 mmol/kg: Gadovist, Bayer Pharma AG, Germany) was administered intravenously prior to inversion recovery sequences. Modified look-locker inversion recovery sequence was used to complete myocardial mapping. ECV was used as an index of myocardial interstitial fibrosis [4] and was calculated as:

$$ECV=\left(\triangle R1_{myocardium}/\triangle R1_{blood-pool}\right)\times\left(1-haematocrit\right)$$, where:

$\triangle R1=\left(1/post-contrast\;T1-1/native\;T1\right)$, where post-contrast T1 mapping has been histologically validated in endomyocardial biopsies to assess interstitial myocardial fibrosis [21] and is highly reproducible [22].

Left ventricular mass was indexed (LVMI) to body surface area, as previously described [20]. Cardiac output was also measured using CMR.

### Data analysis

Autonomic testing data was exported from LabChart and imported into a data analysis program (Spike 8, Cambridge Electronic Design Limited, Cambridge, UK). MSNA was quantified as burst frequency (bursts per minute) and burst incidence (bursts per 100 heartbeats). Bursts were identified using a script, with a detection amplitude set 2 standard deviations above noise [16] and manually confirmed. Every R wave was then marked in the ECG channel and the latency between R waves and subsequent MSNA bursts was calculated and checked (~ 1.3 s). Baseline MSNA, beat-to-beat heart rate and blood pressure were averaged from the 5–10-min baseline. For the CPT, data were averaged over the 2-min rest period and between 60 and 90 s of the CPT (peak). The absolute and percentage change from rest to peak CPT were calculated.

### Statistical analyses

All data were analysed using SPSS statistics (V28.0, IBM, New York, USA). Participant characteristics, baseline data (including CMR data) and absolute and percentage change from rest to peak CPT data were analysed using an independent samples *t*-test or Mann–Whitney *U* test if data were not normally distributed. The time course of the CPT was analysed using a two-way mixed-model ANOVA, with rest and peak CPT as the 2 time points, and normotensive and hypertensive participants as the 2 groups. Linear regression (enter method) with ECV as the dependent variable was completed to examine independent associations with MSNA, MSNA reactivity to the CPT and daytime ABPM. Models with resting MSNA and MSNA reactivity were adjusted for daytime systolic blood pressure (SBP) as a possible confounder. Data are reported as mean ± standard deviation or median [interquartile range]. Alpha was set at 0.05.

## Results

### Participants

**Fig. 1:**
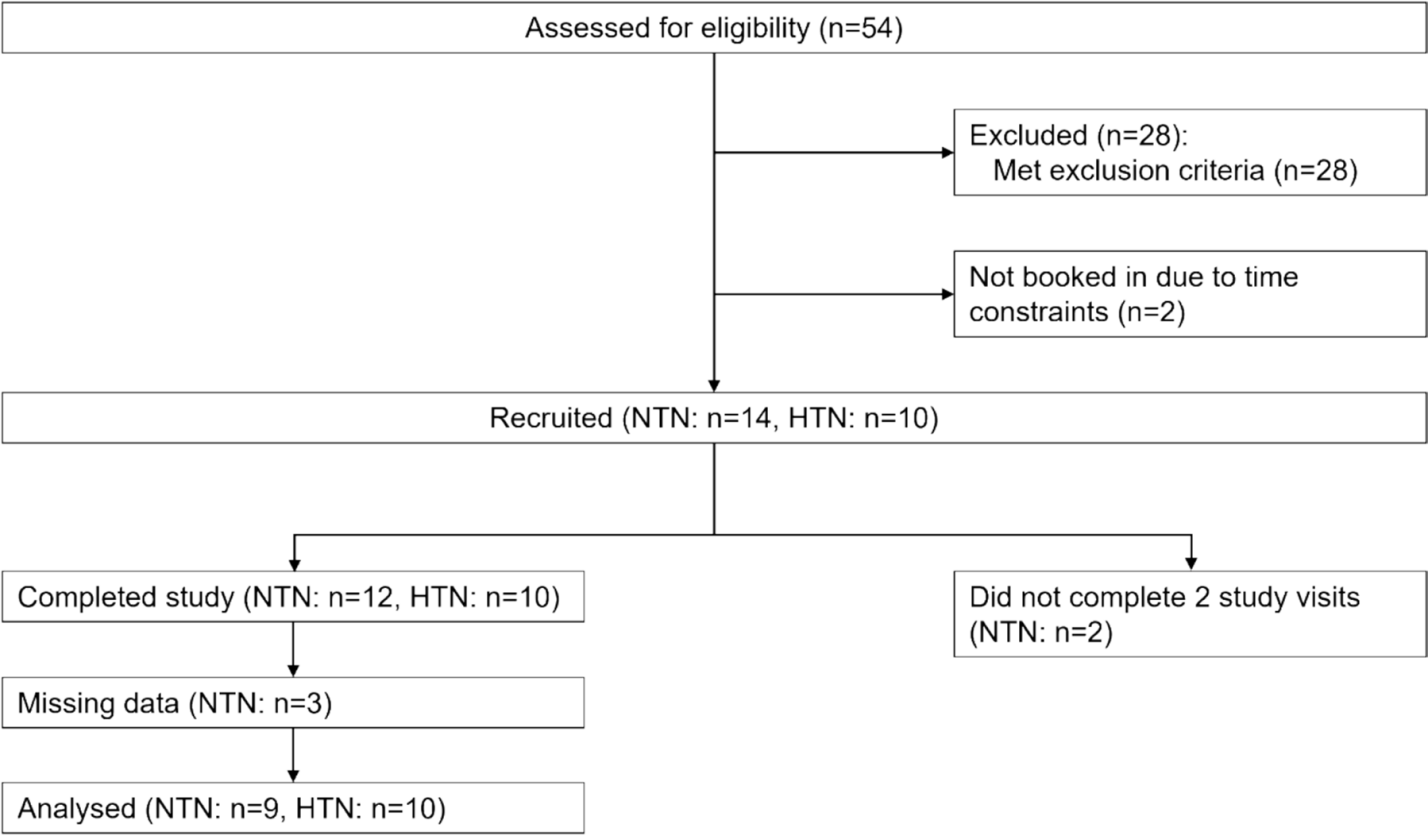
Participant recruitment diagram. CONSORT diagram showing the number of participants screened, recruited and completed the study. NTN normotensive, HTN hypertensive. A priori power analysis was conducted for sample size

A CONSORT diagram showing study recruitment is shown in Fig. 1. Fifty-four participants received a participant information sheet and were screened for this study; however, 28 were excluded due to screening failure (see Additional file 1: Table S1 for details). Twenty-four participants were recruited (normotensive *n* = 14, hypertensive (grade 1 [14]) *n* = 10); however, two normotensive participants did not return for the CMR visit, and three data sets were incomplete, thus were not included in the analyses.

The final sample size was 9 normotensive (4 male) and 10 hypertensive (6 male) participants. Participant characteristics and medications are shown in Table 1. Mean age, BMI and body surface area were similar between hypertensive and normotensive participants. Participants also had similar levels of cardiopulmonary fitness (based on VO_2_ peak; *P* = 0.454). The age range was 29–65 years and 27–64 years for the normotensive and hypertensive groups respectively. Two hypertensive participants were untreated, two were treated with controlled blood pressure, and six were treated with blood pressure above target (> 135/85 mmHg daytime ABPM). The number and class of antihypertensive medications used by hypertensive participants is shown in Table 1. Four normotensive and three hypertensive participants were post-menopausal. Hormonal contraception was not used by any participant.

**Table 1:** Participant characteristics and prescribed medications

	NTN (*n* = 9)	HTN (*n* = 10)	*P* value	Effect size Cohen’s *D*
Age (years)	47 ± 14	49 ± 12	0.679	0.194
Sex (M/F)	5/4	6/4		
Height (cm)	172.2 ± 10.1	175.7 ± 11.7	0.500	0.316
Weight (kg)	78.2 ± 13.3	82.7 ± 11.2	0.436	0.367
BMI (kg/m^2^)	26.3 ± 2.9	26.8 ± 2.5	0.696	0.182
BSA (m^2^)	1.93 ± 0.21	2.01 ± 0.19	0.417	0.382
VO_2_ peak (ml/min/kg)	24.6 ± 6.4	22.4 ± 5.0	0.454	0.388
Clinic SBP (mmHg)	125 ± 8	143 ± 18	**0.011**	1.318
Clinic DBP (mmHg)	80 ± 5	90 ± 7	**0.002**	1.693
Clinic HR (bpm)	74 ± 13	75 ± 11	0.799	0.119
Daytime SBP (mmHg)	119 ± 8	133 ± 13	**0.013**	1.273
Daytime DBP (mmHg)	77 ± 5	89 ± 9	** < 0.001**	2.134
Antihypertensive medications				
ACE inhibitor	0	6	–	–
ARB	0	1	–	–
CCB	0	3	–	–
β-Blocker	0	1	–	–
Other medications				
SSRIs	1	1	–	–
Statins	1	2	–	–
PPIs	0	2	–	–
Levothyroxine	1	1	–	–

Data are mean ± standard deviation

*NTN* normotensive, *HTN* hypertensive, *M* male, *F* female, *BMI* body mass index, *BSA* body surface area, *SBP* systolic blood pressure, *DBP* diastolic blood pressure, *HR* heart rate, *bpm* beats per minute, *ACE* angiotensin converting enzyme, *ARB* angiotensin receptor blocker, *CCB* calcium channel blocker, *SSRIs* selective serotonin reuptake inhibitors, *PPI* proton pump inhibitor. Independent samples *t*-test

Significant values are in bold

### Resting neural-haemodynamic and cardiac parameters

Clinic and ambulatory SBP and DBP were higher in the hypertensive group compared to normotensive controls (Table 1). Resting neural-haemodynamic and cardiac MRI variables are shown in Table 2. Baseline beat-to-beat SBP and DBP measured via the Finapres during quiet rest were similar between groups. Baseline MSNA expressed as burst frequency (bursts per minute) was higher in the hypertensive versus the normotensive group, but MSNA was similar between groups when expressed as bursts/100 heartbeats. ECV, an early marker of myocardial interstitial fibrosis [23, 24], was not different between groups and was within the previously reported normal reference ranges [25]. Cardiac output (measured via CMR) was also similar between groups.

**Table 2:** Baseline neural-haemodynamic and cardiac MRI variables during quiet rest

	NTN (*n* = 9)	HTN (*n* = 10)	*P* value	Effect size Cohen’s *D*
Bf (bursts/min)	36 ± 5	45 ± 9	**0.017**	1.217
BI (bursts/100 Hb)	59 ± 8	67 ± 10	0.088	0.832
HR (bpm)	61 ± 7	67 ± 11	0.180	0.643
SBP (mmHg)	141 ± 21	150 ± 13	0.277	0.517
DBP (mmHg)	74 ± 11	80 ± 6	0.139	0.713
CMR cardiac output (L/min)	5.6 ± 0.9	6.2 ± 1.4	0.306	0.485
Ejection fraction (%)	60 ± 3	60 ± 3	0.889	0.065
ECV (%)	25.7 ± 4.0	24.4 ± 2.0	0.393	0.402
LVM (g)	105 ± 17	109 ± 28	0.720	0.188
LVM index (g/m^2^)	55 ± 8	55 ± 13	1.000	0.000

Data are mean ± standard deviation

*NTN* normotension, *HTN* hypertension, *Bf* burst frequency, *BI* burst incidence, *Hb* heartbeats, *HR* heart rate, *bpm* beats per minute, *SBP* systolic blood pressure, *DBP* diastolic blood pressure, *CMR* cardiac magnetic resonance imaging, *ECV* extracellular volume, *LVM* left ventricular mass. Independent samples *t*-test and Mann-Whitney *U* test (LVM and LVM index)

Significant values are in bold

### Sympathetic and haemodynamic responses to the cold pressor test

**Fig. 2:**
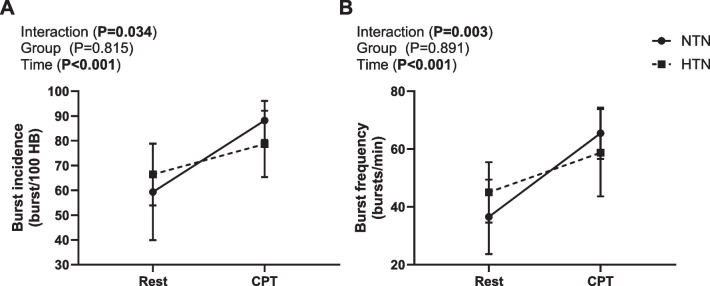
Average sympathetic responses to CPT. Mean ± SD **A** burst incidence and **B** burst frequency, in normotensive (NTN: *n* = 9, circle) and hypertensive (HTN: *n* = 10, square) participants. Two-way mixed-model ANOVA, Bonferroni post hoc. Data are averaged 2-min rest vs peak of cold pressor test (second 30 s)

To examine dynamic sympathetic, BP and heart rate responses to a physiological stressor, the CPT was used. The MSNA response to the CPT was different between groups (MSNA expressed as bursts/100 heartbeats (burst incidence): time*group interaction *P* = 0.034, and MSNA expressed as bursts/min (burst frequency): time*group interaction *P* = 0.003, Fig. 2). The normotensive group had a greater change in burst incidence and burst frequency from rest to peak CPT (NTN: 29 ± 20 bursts/100 heartbeats versus HTN: 12 ± 10 bursts/100 heartbeats, *P* = 0.034, NTN: 29 ± 10 bursts/min versus HTN: 14 ± 8 bursts/min, *P* = 0.003, Additional file 1: Fig. S1), compared to the hypertensive group.

**Fig. 3:**
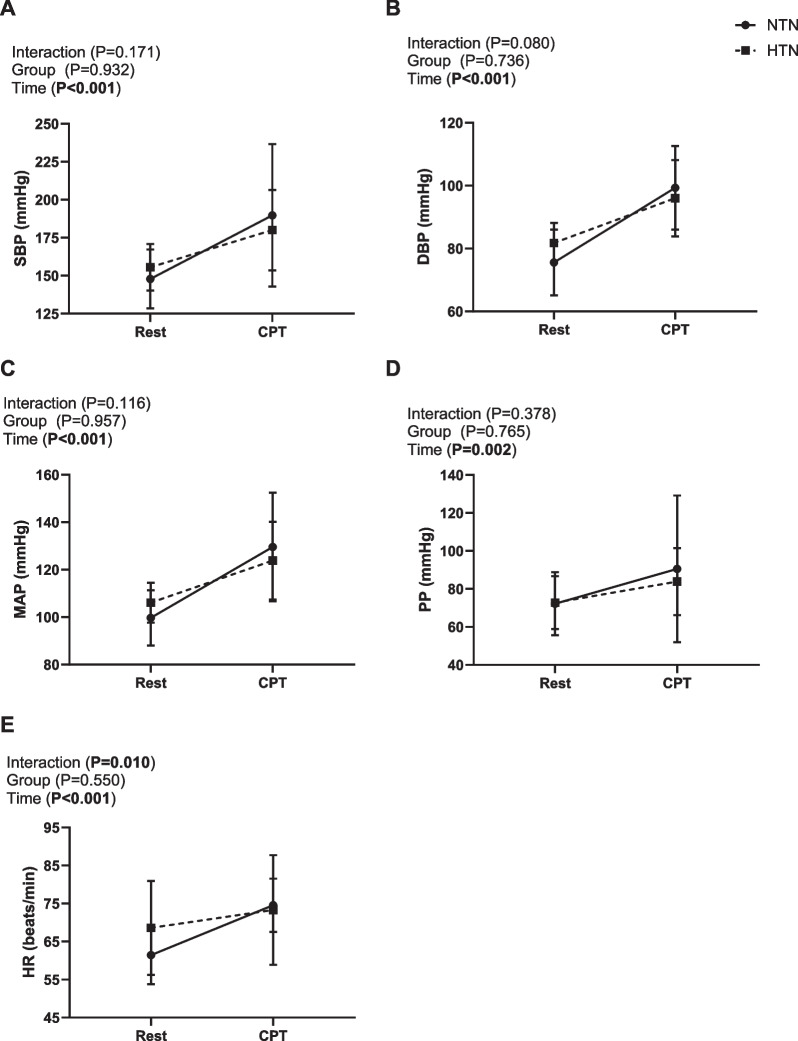
Average cardiovascular responses to the CPT. Mean ± SD **A** systolic blood pressure (SBP), **B** diastolic blood pressure (DBP), **C** mean arterial pressure (MAP), **D** pulse pressure (PP) and **E** heart rate (HR) in normotensive (NTN: *n* = 9, circle) and hypertensive (HTN: *n* = 10, square) participants. Two-way mixed-model ANOVA, Bonferroni post hoc. Data are averaged 2-min rest vs peak of cold pressor test (second 30 s)

There was no difference in the blood pressure response to the CPT between groups (time*group interaction *P* > 0.05, Fig. 3, for exact *P* values). SBP, DBP, MAP and PP increased from rest to peak CPT (Fig. 3). The change from rest to peak CPT in SBP, DBP and PP were similar between groups (*P* > 0.05, Additional file 1: Fig. S2); however, the normotensive group had a greater increase in MAP compared to the hypertensive group (Additional file 1: Fig. S2).

There was a difference in the HR response to the CPT between groups (time*group interaction *P* = 0.010, Fig. 3), with the normotensive group having a greater increase in HR from rest to peak CPT, compared to the hypertensive group. Accordingly, the change in heart rate from rest to peak CPT was higher in the normotensive group compared to the hypertensive group (*P* < 0.05, for exact *P* values, Additional file 1: Fig. S3).

### Sympathetic nerve activity and extracellular volume

**Fig. 4:**
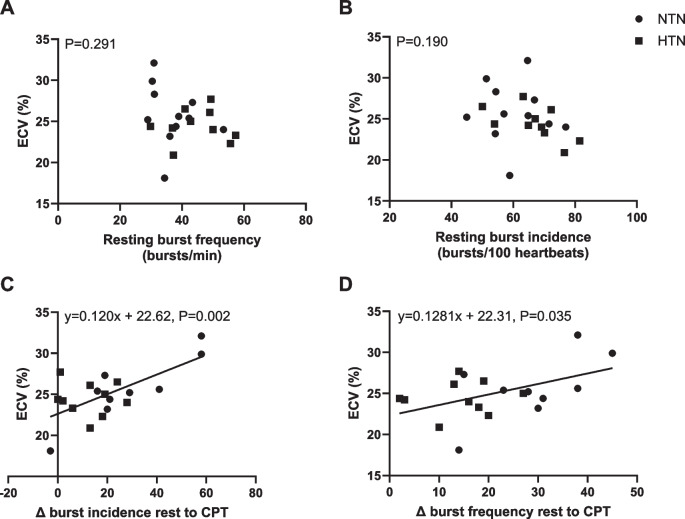
Unadjusted linear regressions between muscle sympathetic nerve activity (MSNA) and extracellular volume (ECV) in people with and without hypertension. No associations were found between ECV and **A** resting burst frequency or **B** resting burst incidence. Significant positive associations observed between ECV and **C** change in burst incidence from rest to peak cold pressor test (CPT) and **D** change in burst frequency, in normotensive (NTN, circle) and hypertensive (HTN, square) individuals (*N* = 19)

To assess whether MSNA was associated with the level of ECV, a multiple linear regression was completed and adjusted for blood pressure level with all participants grouped together (Table 3). Resting MSNA (burst incidence) was not related to the level of ECV (*B* coefficient = − 0.007, CI − 0.24–0.10, *P* = 0.549); however, the change in MSNA from baseline to the CPT was related to the level of ECV (*B* coefficient = 0.12, CI 0.050–0.20, *P* = 0.007). Thus, for every increase in 1 burst/100 heartbeats of MSNA, the ECV was 0.12% higher. For participants whose MSNA increased by 30 bursts/100 heartbeats (average increase in this study), ECV would be 3.6% higher than those who do not get an increase in MSNA. Unadjusted linear regressions are shown in Fig. 4.

**Table 3:** Linear regression between muscle sympathetic nerve activity (MSNA) and extracellular volume (ECV)

	Unadjusted (*n* = 19)				Adjusted* (*n* = 19)			
	*B* coefficient (95% CI)	*R*	*R*^2^	*P* value	*B* coefficient (95% CI)	*R*	*R*^2^	*P* value
Resting MSNA (bursts/100 heartbeats)	− 0.08 (− 0.24–0.08)	0.25	0.06	0.302	− 0.07 (− 0.24–0.10)	0.27	0.07	0.549
∆MSNA during CPT (bursts/100 heartbeats)	0.12 (0.05–0.19)	0.68	0.46	**0.002**	0.12 (0.05–0.20)	0.68	0.46	**0.007**
Daytime SBP	− 0.04 (− 0.16–0.08)	0.17	0.03	0.485	–	–	–	–

*Adjusted for daytime systolic ambulatory blood pressure monitoring. *CPT* cold pressor test, *SBP* systolic blood pressure

Significant values are in bold

## Discussion

The key findings of this study were that (1) MSNA at rest is not associated with MRI measures of ECV, however, the increase in SNA during a stressor predicts the ECV in people with hypertension and normal BP levels, indicating that irrespective of daytime ambulatory BP, surges in SNA following a stressor are associated with higher levels of ECV, and (2) the sympathetic response to a physiological stressor was blunted in hypertensive versus normotensive individuals, despite the SBP and DBP responses being similar between groups.

Resting MSNA was not associated with ECV in the present study. There is no research investigating whether MSNA is associated with ECV in humans at rest. One study showed that excision of the superior cervical ganglion in SHRs with known elevated SNA attenuated cardiac fibrosis, compared to SHRs with an intact superior cervical ganglion [11]; however, since the superior cervical ganglion does not play a major functional role in SNA innervation of the heart [26], this does not necessarily indicate that SNA directed at the myocardium is the cause of myocardial fibrosis. Although MSNA is not a measure of cardiac sympathetic drive and reflects the sympathetic outflow to skeletal muscle vasculature, it has been demonstrated to parallel cardiac noradrenaline spillover [27].

We found that in all participants, SNA reactivity (change in MSNA from rest to peak CPT) predicted ECV, an early marker of myocardial interstitial fibrosis, where the greater the MSNA response to the CPT, the higher the ECV, independent of ambulatory BP level. To our knowledge, this is the first evidence that sympathetic hyperactivity to a physiological stressor is a predictor of ECV in humans. Previous studies have shown that acute stress can drive cardiac damage, such as in Takotsubo cardiomyopathy, where stress-induced catecholamine overload is a proposed important factor for its development [28]. Supporting our findings, previous research has shown that overactivation of β-adrenergic receptors in mice promoted cardiac fibrosis [13, 29]. Importantly, Ma and colleagues [13] revealed that intermittent stimulation of the β-adrenergic receptors with isoprenaline resulted in more severe cardiac fibrosis compared to sustained β-adrenergic receptor stimulation with the same dose. We showed that overall resting MSNA was not associated with ECV; however, surges in MSNA in response to a stressor were positively associated with ECV, independent of resting ambulatory blood pressure, and blood pressure reactivity. This is important considering we know that elevated resting levels of MSNA contribute to the development of hypertension [30–32], but it cannot be determined whether MSNA contributed to the development of interstitial fibrosis in the present study. Our study shows for the first time that SNA reactivity is important in predictive ECV. If there is a causal link between SNA and ECV, one possible explanation is that intermittent surges in SNA following a stressful stimulus may increase collagen and fibrin production in the extracellular space, resulting in raised ECV. Additionally, it is possible that greater sympathetic surges promote higher ECV because this leads to exacerbated release of noradrenaline and the co-transmitter neuropeptide Y (NPY) from the sympathetic nerve endings. Combined, this could potentiate the negative effects of beta-adrenergic receptor stimulation on the myocardium recruiting more fibroblasts and greater macrophage infiltration [29, 33].

Along these lines, the association may also be explained by an acute inflammatory response to the CPT, as inflammation plays a pivotal role in cardiac fibrosis development [34]. Accordingly, it has previously been shown that levels of inflammatory regulators intracellular adhesion molecule (ICAM-1) and vascular cell adhesion molecule (VCAM-1) were upregulated immediately after the CPT in people with and without hypertension, compared to resting levels [35, 36]. With inflammation being associated with elevated ICAM-1 and VCAM-1 levels, it is possible that an acute local inflammatory response to the CPT occurs. This may contribute to cardiac fibrosis development via noradrenaline binding to α-adrenergic receptors expressed on macrophages, ultimately increasing macrophage activity [37]. Inflammatory markers were not measured pre- and post-CPT, so an acute inflammatory response remains speculative.

The hypertensive group had a reduced sympathetic response to the stressor compared to the normotensive group in the present study, despite both groups having a similar SBP and DBP response. This could be due to antihypertensive treatment in the hypertensive group which may blunt BP changes in response to stress. This seems unlikely as it was previously shown that effective antihypertensive therapy (where resting BP is reduced below target levels) does not dampen BP reactivity to metaboreflex isolation [38]. Moreover, previous work showed that MSNA responses to the CPT were reduced in hypertensive subjects without antihypertensive medication, compared to normotensive subjects [39]. Additionally, the CPT is a model of pain, which activates peripheral nociceptors and the SNS [40]; thus, it is possible that the lower sympathetic response to the CPT in hypertensive individuals is a result of a higher pain threshold in this cohort [41–44]. Pain perception was not measured in this study; therefore, this is speculative.

It is important to note that due to the sample size, confounding variables such as lifetime stress exposure, antihypertensive medication and BMI were not adjusted for in the linear regression. It is widely accepted that physical factors such as elevated BMI are strongly associated with adverse cardiovascular outcomes, including increased myocardial fibrosis [45]; however, most research showing associations are conducted in obese individuals with a BMI ≥ 30 kg/m^2^ [45, 46]. Similarly, it has been shown that overweight healthy individuals (BMI: 25.0–29.9 kg/m^2^) have an exaggerated sympathetic reactivity in response to the CPT [47]; however, in the current study, there was no relationship between BMI and sympathetic reactivity (Δ BI (rest to CPT) vs BMI: *y* = − 0.04*x* + 27.36, *P* = 0.257; Δ Bf (rest to CPT) vs BMI: *y* = − 0.09*x* + 28.40, *P* = 0.09). As the average BMI in the study was < 29.9 kg/m^2^ and no relationship between BMI and SNA reactivity was observed, it is unlikely that adding BMI as a confounding variable would influence the relationship.

Finally, the participants with hypertension in this study did not have an elevated ECV compared to the normotensive participants, which may be a result of antihypertensive medications. For example, both ACE inhibitors [48, 49] and angiotensin receptor blockers [50] have been shown to regress interstitial fibrosis in patients with hypertension and increased collagen volume fraction.

### Limitations

A key limitation of this study is the low number of participants; however, associations were observed despite the reduced sample size. As a result of the sample size, it was not possible to add confounding variables into the regression analyses. Furthermore, the participants with hypertension were restricted to stage 1 hypertension; thus, it is not known if similar associations with change in MSNA and ECV would be observed in individuals with more severe hypertension. Additionally, the data collected during this study represents one point in time, therefore cannot provide a definite cause-and-effect relationship. Other physiological stressors, for example mental stress or handgrip exercise, were not assessed; thus, it may be necessary to determine if the association remains with other stressors which are more applicable to daily life. The CPT was chosen as a physiological stressor as it has previously been shown to determine future hypertension [51–53], elicits large increases in BP and MSNA, and is a common stress test used to evaluate sympathetic neural control of the peripheral circulation [54]. However, a limitation of the CPT is that it activates nociceptors, which may induce changes in breathing patterns and subsequent changes in MSNA, HR and BP as a result. A limitation of using ECV as a measure of cardiac fibrosis is that ECV could be higher in some people because they have a greater cardiac mass, rather than increased collagen volume; however, in this study we found no correlation between LV mass and ECV (*r* = 0.34, *P* = 0.105). Finally, MSNA is a measure of sympathetic outflow to skeletal muscle vasculature and is not cardiac specific.

## Conclusions

This study highlights greater increases in SNA in response to a stressor predict the level of ECV, irrespective of hypertension status or resting daytime BP, but that the resting level of SNA does not. This suggests a potentially important role of SNA hyperreactivity to a stressor in determining end organ damage and subsequent cardiovascular risk. Future research determining the mechanisms driving the association between SNA reactivity and ECV may prove beneficial for therapeutic targeting, as humans regularly experience physiological stressors. For example, determining whether repeated surges of sympathetic overactivation are increasing inflammatory markers via β-adrenergic and/or NPY1 receptor stimulation, and subsequently promoting cardiac fibrosis, as demonstrated in animal models [29]. Interventions could be developed to assess and mitigate this excessive stress response to prevent fibrotic processes in the heart that contribute to a multitude of cardiac diseases (e.g. heart failure with preserved ejection fraction, atrial fibrillation).

In summary, our data shows a significant positive association between sympathetic hyperactivity to a stressor and interstitial myocardial fibrosis may exist. It is possible that intermittent surges in SNA play a more important role in cardiac fibrosis development, than overall level of resting SNA; however, future studies are required to investigate this in larger cohorts.

## Supplementary Information

Additional file 1: Contains supplementary methods, including participant inclusion and exclusion criteria, and supplementary results including reasons for screen failure and absolute and percentage change in MSNA, blood pressure and haemodynamic data during the CPT. Fig. S1 Absolute and percentage change in sympathetic responses from rest to peak CPT. Fig. S2 Absolute and percentage change in blood pressure from rest to peak CPT. Fig. S3 Absolute and percentage change in heart rate from rest to peak CPT.

## Data Availability

No datasets were generated or analysed during the current study.

## Abbreviations

ABPM: Ambulatory blood pressure monitor
Bf: Burst frequency
BI: Burst incidence
BMI: Body mass index
BP: Blood pressure
BSA: Body surface area
CMR: Cardiac magnetic resonance imaging
CPET: Cardiopulmonary exercise test
CPT: Cold pressor test
DBP: Diastolic blood pressure
ECV: Extracellular volume
HR: Heart rate
HTN: Hypertension
ICAM-1: Intracellular adhesion molecule-1
LV: Left ventricular
LVH: Left ventricular hypertrophy
LVM: Left ventricular mass
MAP: Mean arterial pressure
MSNA: Muscle sympathetic nerve activity
NPY: Neuropeptide Y
NTN: Normotensive
PP: Pulse pressure
SBP: Systolic blood pressure
SHR: Spontaneously hypertensive rat
SNA: Sympathetic nerve activity
SNS: Sympathetic nervous system
VCAM-1: Vascular cell adhesion molecule-1
