# Supplementary material for: Sympathetic reactivity to physiological stress is associated with expanded cardiac extracellular volume in humans
Source: BMC Med. 2025 Jul 1;23:367. doi: 10.1186/s12916-025-04197-6 (PMC12220307; doi:10.1186/s12916-025-04197-6)
Supplement: Supplementary file 1 — Additional file 1: Contains supplementary methods, including participant inclusion and exclusion criteria, and supplementary results including reasons for screen failure and absolute and percentage change in MSNA, blood pressure and haemodynamic data during the CPT. Fig. S1 Absolute and percentage change in sympathetic responses from rest to peak CPT. Fig. S2 Absolute and percentage change in blood pressure from rest to peak CPT. Fig. S3 Absolute and percentage change in heart rate from rest to peak CPT. [file 12916_2025_4197_MOESM1_ESM.docx]

Sympathetic hyperactivity to physiological stress is associated with expanded cardiac extracellular volumes in humans.

Hazel C Blythe^1*^, Zoe H Adams^1,6^, Katrina A Hope^1^, Richard P Baker^1,2^, Melanie J Hezzell^3^, M. Saadeh Suleiman^4^, Ana Paula Abdala Sheikh^1^, Nathan Manghat^5^, Konstantina Mitrousi^5^, Angus K Nightingale^#1,5^ and Emma C Hart^#1^

^*^ Corresponding author

^#^ Contributed equally to senior authorship of this manuscript

^1^ School of Physiology, Pharmacology and Neuroscience, University of Bristol, Bristol, UK.

^2^ Department of Cardiology, Musgrove Park Hospital, Somerset NHS Foundation Trust, Taunton, UK.

^3^ Bristol Veterinary School, University of Bristol, Bristol, UK.

^4^ Bristol Medical School, University of Bristol, Bristol, UK.

^5^ Bristol Heart Institute, University Hospitals Bristol and Weston NHS Foundation Trust, Bristol, UK.

^6^ Cardiff School of Sport and Health Sciences, Cardiff Metropolitan University, Cardiff, UK.

**Article type:** Research article

**Corresponding author**

Dr Hazel C Blythe

School of Physiology, Pharmacology and Neuroscience

Biomedical Sciences Building

University of Bristol

BS8 1TD

Email: hazel.blythe@bristol.ac.uk

Supplementary materials

**Methods**

Inclusion criteria

The following inclusion criteria were used for,

*All participants:*

1. Aged 25-80 years

*Normotensive participants:*

1. Office blood pressure < 140/90 mmHg and daytime ambulatory blood pressure < 135/85 mmHg

*Hypertensive participants:*

1. Office blood pressure ≥ 140/90 mmHg and daytime ambulatory blood pressure ≥ 135/85 mmHg

Exclusion criteria

The following exclusion criteria were used for all participants:

1. Secondary causes of hypertension
2. Body mass index ≥30 kg/m^2^
3. Pregnancy or breastfeeding women
4. Taking nitrate, steroid or immunosuppressant medication, or use of medication as part of a clinical trial
5. Major illness such as cancer, inflammatory disease (including vasculitis) or receiving palliative care
6. Diagnosed cardiovascular (including stroke), respiratory (including chronic obstructive pulmonary disease), psychiatric, renal or ophthalmic disease
7. Congenital or acquired neurological conditions (including dementia), language disorders, repeated or chronic pain conditions (excluding menstrual pain and minor sporadic headaches)
8. Metabolic or endocrine disorders such as diabetes mellitus (type I and II)
9. Symptoms of febrile illness 2 weeks prior to experiments
10. Excessive alcohol consumption (>28 units/week) or use of illicit drugs
11. Heart transplant
12. Coronary revascularisation
13. Heart failure-related hospitalisation

Participants VO_2peak ­_was assessed using an incremental exercise test on an upright cycle ergometer (Ergoselect 100, Love Medical, UK). Cardiorespiratory data were recorded using the CPET system (Ergostik, Love Medical, UK), with integrated 12-lead ECG, pulse oximetry and automated blood pressure cuff to monitor heart rate, oxygen saturations and blood pressure respectively. VO_2peak_ data was averaged over the final 30 seconds of exercise.

Studentised residuals for SBP, MAP and burst incidence were not normally distributed, and no transformation rectified this, thus raw data was used. PP failed Levene’s test however no transformation rectified this, thus raw data was used.

**Results**

Twenty-eight individuals were excluded due to screening failure (Supplementary Table 1).

Table S1. Reasons for screening failure

| Exclusion criteria | Number of individuals meeting exclusion criteria |
| --- | --- |
| Neurological conditions | 2 |
| Diabetes mellitus | 2 |
| Immunosuppressants / steroids / medication as part of a clinical trial | 6 |
| BMI >30kg/m^2^ | 11 |
| Undergoing cardiovascular monitoring | 1 |
| Use of recreational drugs | 1 |
| Diagnosed with long COVID | 1 |
| Age | 1 |
| Cardiovascular disease | 3 |

Figure S1. Absolute and percentage change in sympathetic responses from rest to peak CPT

Mean ± SD or median [IQR] (A) absolute and (B) percentage change in burst incidence and (C) absolute and (D) percentage change in burst frequency, in normotensive (NTN: n=9, blue) and hypertensive (HTN: n=10, red) participants. Independent samples t-test or Mann Whitney U test, rest to peak CPT.

Figure S2. Absolute and percentage change in blood pressure from rest to peak CPT

Mean ± SD or median [IQR] (A) absolute and (B) percentage change in systolic blood pressure, (C) absolute and percentage (D) change in diastolic blood pressure, (E) absolute and (F) percentage change in mean arterial pressure, and (G) absolute and (H) percentage change in pulse pressure in normotensive (NTN: n=9, blue) and hypertensive (HTN: n=10, red) participants. Independent samples t-test or Mann Whitney U test, rest to peak CPT.

Figure S3. Absolute and percentage change in heart rate from rest to peak CPT

Mean ± SD (A) absolute and (B) percentage change in heart rate in normotensive (NTN: n=9, blue) and hypertensive (HTN: n=10, red) participants. Independent samples t-test, rest to peak CPT.
